# Virtual biopsy through CT imaging: can radiomics differentiate between subtypes of non-small cell lung cancer?

**DOI:** 10.1007/s11547-025-02022-x

**Published:** 2025-05-22

**Authors:** Federica Palmeri, Marta Zerunian, Michela Polici, Stefano Nardacci, Chiara De Dominicis, Bianca Allegra, Andrea Monterubbiano, Massimiliano Mancini, Riccardo Ferrari, Pasquale Paolantonio, Domenico De Santis, Andrea Laghi, Damiano Caruso

**Affiliations:** 1 Department of Medical-Surgical Sciences and Translational Medicine, School of Medicine and Psychology, Sapienza - University of Rome, Sant’Andrea University Hospital, Rome, Italy; 2 PhD School in Translational Medicine and Oncology, Department of Medical and Surgical Sciences and Translational Medicine, Faculty of Medicine and Psychology, Sapienza University of Rome, Rome, Italy; 3 Computer Science Department, Sapienza University of Rome, Rome, Italy; 4 Morphologic and Molecular Patology Unit, Sant’Andrea University Hospital, Rome, Italy; 5 Emergency Radiology Department, San Camillo-Forlanini Hospital, Rome, Italy; 6 Department of Radiology, San Giovanni Addolorata Hospital Complex, Rome, Italy

**Keywords:** Lung, Non-small-cell lung cancer (NSCLC), Radiomics, Computed tomography (CT), Virtual biopsy

## Abstract

**Objective:**

This study evaluated the performance of CT radiomics in distinguishing between lung adenocarcinoma (ADC) and squamous cell carcinoma (SCC) at baseline imaging, exploring its potential as a noninvasive virtual biopsy.

**Materials and methods:**

A retrospective analysis was conducted, enrolling 330 patients between September 2015 and January 2023. Inclusion criteria were histologically proven ADC or SCC and baseline contrast-enhanced chest CT. Exclusion criteria included significant motion artifacts and nodules < 6 mm. Radiological features, including lung lobe affected, peripheral/central location, presence of emphysema, and T/N radiological stage, were assessed for each patient. Volumetric segmentation of lung cancers was performed on baseline CT scans at the portal-venous phase using 3DSlicer software (v5.2.2). A total of 107 radiomic features were extracted and selected using the least absolute shrinkage and selection operator (LASSO) and tenfold cross-validation. Multivariable logistic regression analysis was employed to develop three predictive models: radiological features-only, radiomics-only, and a combined model, with statistical significance set at *p* < 0.05. Additionally, an independent external validation cohort of 16 patients, meeting the same inclusion and exclusion criteria, was identified.

**Results:**

The final cohort comprised 200 ADC and 100 SCC patients (mean age 68 ± 10 years, 184 men). Two radiological and 21 radiomic features were selected (*p* < 0.001). The Radiological model achieved AUC 0.73 (95% CI 0.68–0.78, *p* < 0.001), 72.3% accuracy. The radiomics model achieved AUC 0.80 (95% CI 0.75–0.85, *p* < 0.001), 75.6% accuracy. The combined model achieved AUC 0.84 (95% CI 0.80–0.88, *p* < 0.001), 75.3% accuracy. External validation (*n* = 15) yielded AUC 0.78 (*p* = 0.05).

**Conclusion:**

The combined radiologic-radiomics model showed the best performance in differentiating ADC from SCC.

## Introduction

Lung cancer is the second most commonly diagnosed cancer in the world, with 2.1 million new cases each year, and the leading cause of cancer death, with an estimated 1.8 million deaths in 2020 [1]. About 85% of lung cancer cases are classified as non-small cell lung cancer, with adenocarcinoma (ADC) and squamous cell carcinoma (SCC) being the most prevalent subtypes [2]. Recent advancements in personalized medicine and targeted medicine, including immunotherapy, have improved the prognosis of lung cancer patients [3] and the unique morphological and molecular traits that distinguish pathological subtypes directly impact therapeutic management and prognosis [4, 5].

Hence, it is critical to characterize pathological subtypes before treatment to develop more effective therapies. Bronchoscopy and surgical biopsy remain the gold standard for molecularly characterizing the tumor. Still, they are invasive and may be associated with potential complications and false-negative results, not being representative of the whole tumor heterogeneity.

In this context, imaging has great potential to guide therapy because it can give a more comprehensive view of the entire tumor in a noninvasive manner [6, 7]. CT is the imaging technique routinely used in cancer management from diagnosis to follow-up; however, the subjective and qualitative imaging assessment has limitations, such as histotype characterization and prognosis. To overcome this, extracting quantitative ultra-structural information from imaging data, with a radiomics approach, might allow for a better characterization of tumor properties. Radiomics is an “omics” branch focused on improving imaging analysis data assessment using a high-throughput extraction of large amounts of quantitative features [8, 9]. The high-dimensional data extraction from digital images is motivated by the fact that biomedical images contain information that reflects pathophysiology and that this correlation can be extrapolated from quantitative analysis [10]. Due to the high incidence of lung cancer, and the critical diagnostic, prognostic, and therapeutic implications, non-small cell lung cancer represents an ideal opportunity for the use of radiomics. Since its inception, lung cancer radiomics has generated substantial research interest, with many published studies in the last years demonstrating a growing interest in the field [11, 12], ranging from determining a nodule’s risk of malignancy [13], to assessing its invasiveness [14], and to predict response to therapy [15, 16].

Therefore, the study aimed to evaluate the performance of radiomics in distinguishing between lung adenocarcinoma and squamous cell carcinoma at baseline imaging evaluation, using radiomics as virtual biopsy.

## Materials and methods

### Patient population and study design

This retrospective observational non-interventional study was conducted in accordance with the 1964 Declaration of Helsinki and its later amendments or comparable ethical standards. Approval was granted by the local ethics boards (CE 6410/2021) as part of the participation at Horizon 2020 “CHAIMELEON - Accelerating the lab to market transition of AI tools for cancer management” (H2020-SC1-FA-DTS-2019-1).

**Fig. 1:**
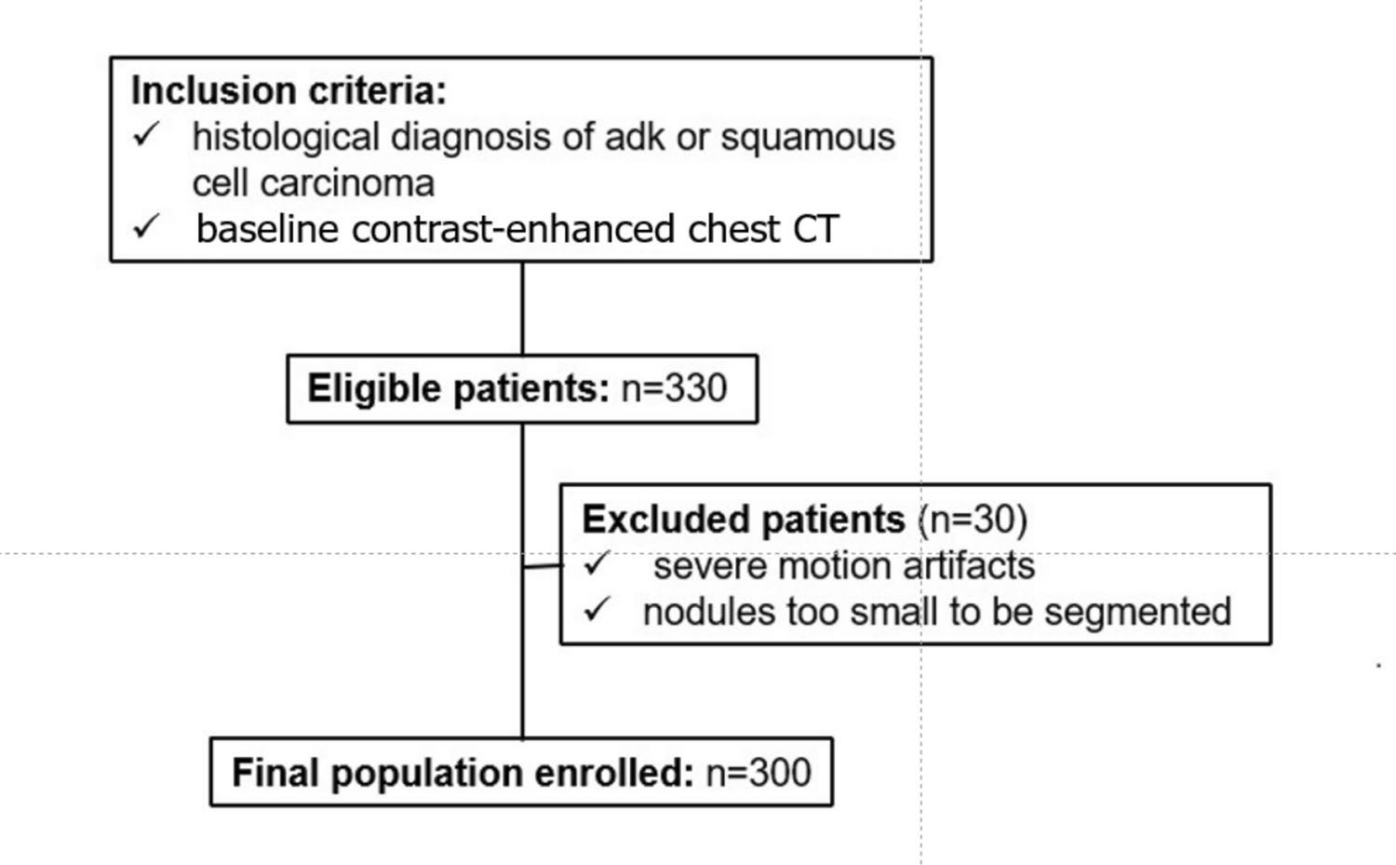
Flowchart shows participant enrollment for model training

From September 2015 to January 2023, 330 consecutive patients who underwent pulmonary resection at Sant’Andrea University Hospital, Rome, Italy were selected according to the following inclusion criteria: (a) proven histological diagnosis of adenocarcinoma or squamous cell carcinoma, and (b) baseline contrast-enhanced chest CT. Exclusion criteria were: (a) significant motion artifacts on chest CT and (b) nodules too small to be segmented (< 6 mm) and with low clinical relevance [17] (Fig. 1). All patients underwent surgical resection within 6–8 weeks of the CT scan used for segmentation, and no chemotherapy or radiation therapy was administered during this period. Therefore, the histology referenced in our study corresponds to the tissue obtained from the surgical specimen. Patient characteristics, including sex and age, and histological reports after biopsy were also retrieved.

### CT acquisition technique

Chest CT was performed using a 128-section CT scanner (Revolution EVO, GE Medical Systems, Milwaukee, USA) with a z-axis coverage from the jugular to the diaphragmatic dome. The following technical parameters were used: tube voltage, 120 kV; tube current modulation, 100–250 mAs with tube current modulation; spiral pitch factor, 0.98; collimation width, 0.625. Images were reconstructed at a slice thickness of 1.25 mm with standard SOFT and LUNG kernels. Contrast medium (Iomeprol 400, Bracco, Milan, Italy) was administered through peripheral venous access of 18/16-gauge. The dose of contrast medium was calculated based on the patient’s lean body weight using a ratio of 700 mg Iodine per kilogram of lean body weight [18]. The injection was monitored through bolus tracking, and the acquisition was performed 50 s after reaching a peak of 150 HU in the region of interest positioned in the aortic arch.

### Radiological assessment

Digital Imaging and Communications in Medicine (DICOM) data were retrieved and analyzed by two radiologists in consensus (CD and MP with 20 and 5 years of experience in thoracic imaging, respectively).

**Fig. 2:**
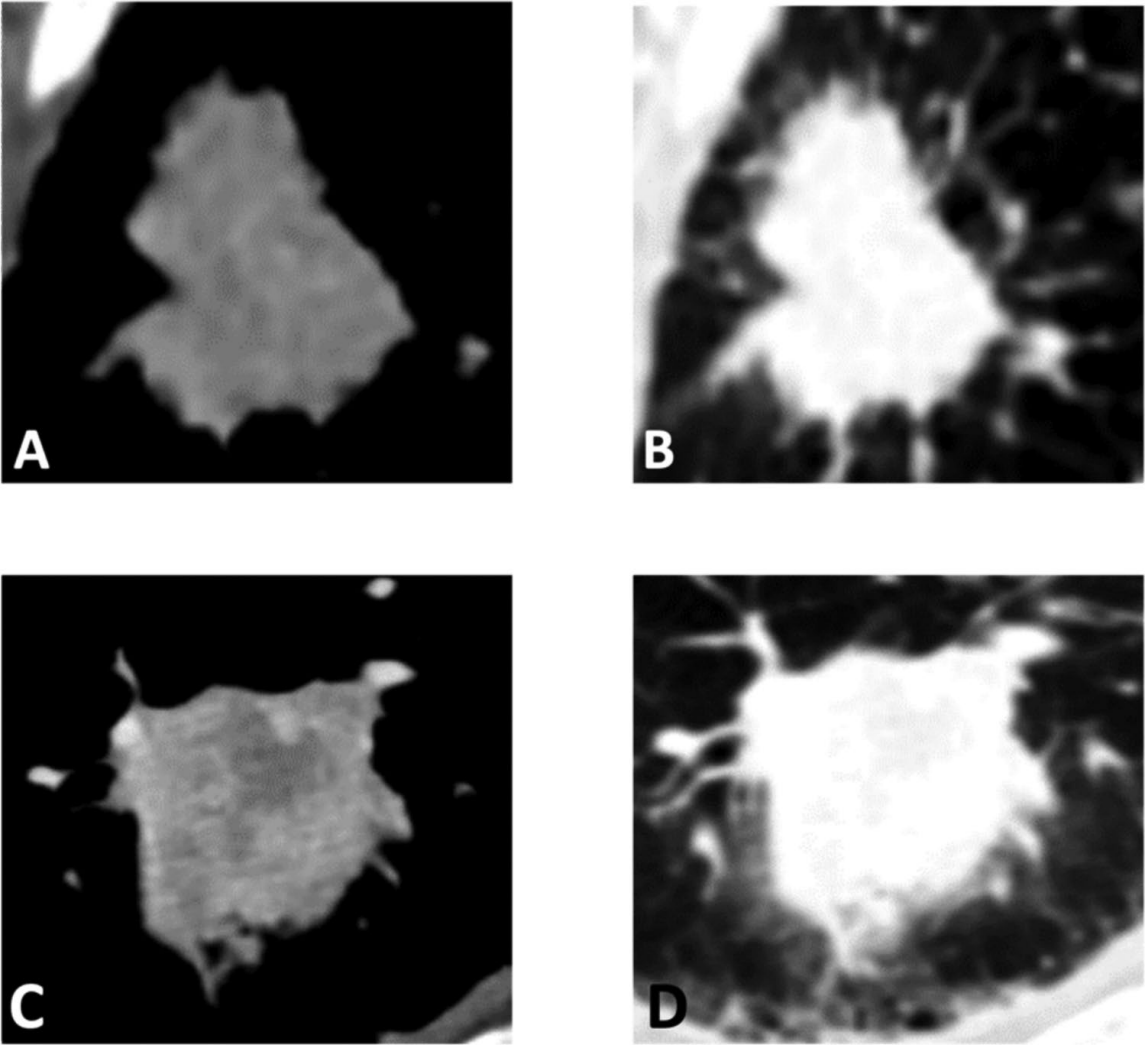
Image in (**A**) mediastinal window and (**B**) lung window of axial thin-section contrast-enhanced chest CT of a lung adenocarcinoma (ADC) in a 65-year-old man. Image in (**C**) mediastinal window and (**D**) lung window of axial thin-section contrast**-**enhanced chest CT of a lung squamous cell carcinoma in a 73-year-old man. The two histological phenotypes do not show substantial differences at CT scans, which would allow them to be differentiated based on their visual features alone

The following image radiological features were assessed: (a) the lung lobe where the lesion was detected; (b) the lesion location within the lobe (central/peripheric); (c) the presence of emphysema; (d) the radiological T and N staging [19–21]. All radiological features except N staging were evaluated on the portal-venous phase, with a SOFT kernel with fixed lung window settings (window width [WW]: 1500 HU, window level [WL]:-600 HU). N staging was assessed on the portal-venous phase, with SOFT kernel, with fixed window settings for the soft tissue of the mediastinum (WW: 350, WL: 50), as shown in Fig. 2.

### Segmentation analysis

DICOM data were retrieved into a multimodality workstation (Centricity Universal Viewer, version 6.0; GE Medical Systems); two radiologists in consensus, DC, and MZ, with 15 years and 5 years of experience in thoracic imaging, respectively, evaluated the CT scans eligible for segmentation analysis according to the inclusion/exclusion criteria. Volumetric lung segmentation of each CT scan was performed using the open-source software 3D Slicer (version 5.2.2, http://www.slicer.org).

**Fig. 3:**
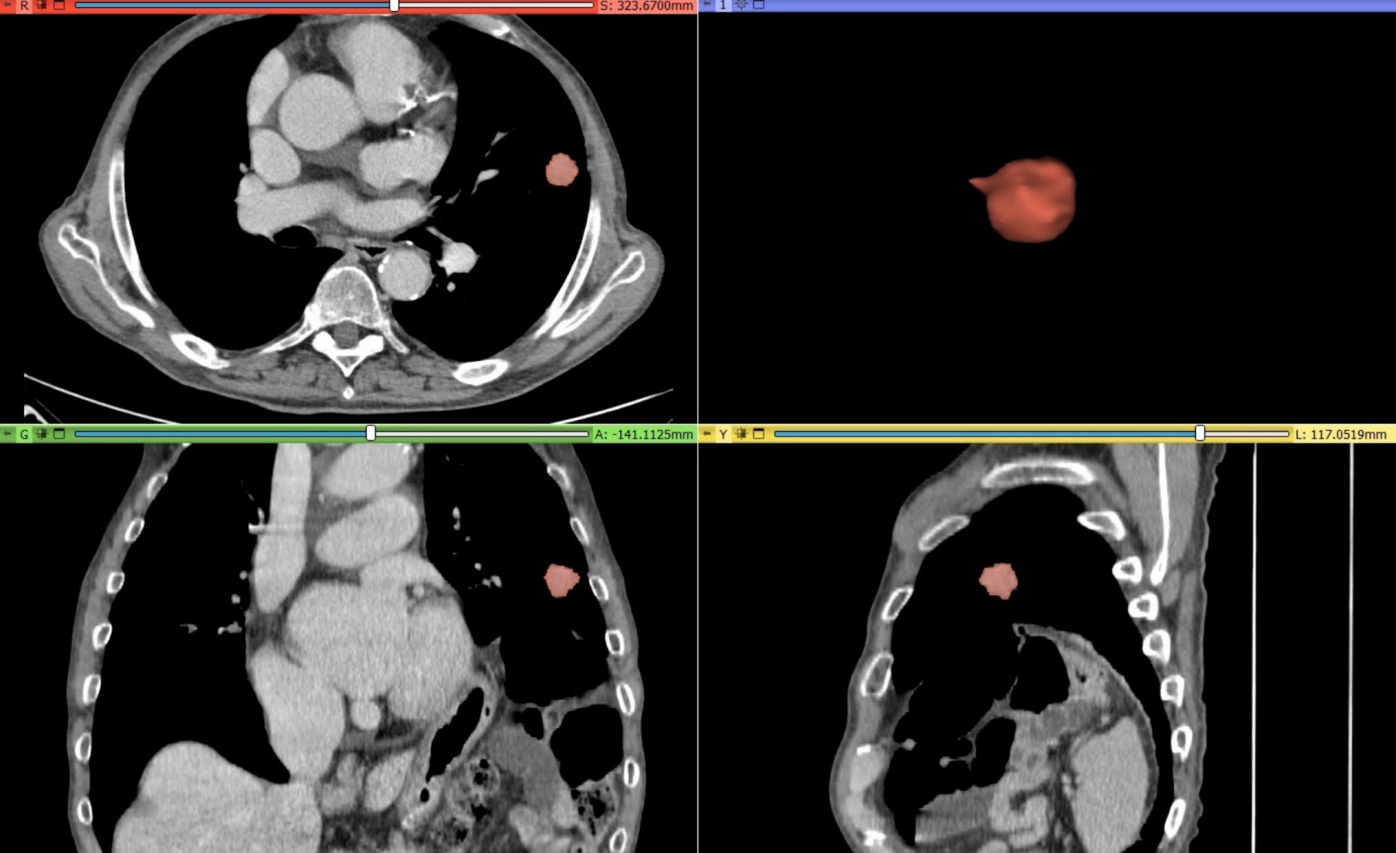
Radiomic volumetric segmentation (in red) of a lung lesion of the upper left lobe of a 55-year-old patient made using the open-source software 3D Slicer (version 5.2.2, http://www.slicer.org)

A volumetric region of interest was manually drawn slice by slice on the SOFT kernel with fixed WWLL for mediastinal tissue as mentioned, avoiding the subsolid areas within the nodules, pulmonary vessels, and bronchi, to cover only the whole solid volume, as shown in Fig. 3.

### Radiomic features extraction

3D Slicer radiomics extension (Pyradiomics [22]) was used to extract 107 radiomics features from the mediastinal window of contrast-enhanced chest CT scans, including first- and second-order features: 19 features first-order statistics, 13 features 2D and 3D shape, 16 features gray-level size zone matrix (GLSZM), 5 features neighboring gray tone difference matrix (NGTDM), 14 features gray-level dependence matrix (GLDM), 24 features gray-level co-occurrence matrix (GLCM), and 16 features gray-level run length matrix (GLRLM).

### Machine learning modeling and statistical analysis

Continuous data are expressed as mean ± standard deviation (SD); categorical variables are reported as counts and percentages. Continuous parametric variables were compared using the Student’s t-test, whereas nonparametric variables were analyzed with the Mann–Whitney U test. To select CT radiomics features, the machine learning (ML) model was developed starting from the initial identification of the radiomics features with near-zero and zero variance; then highly correlated features were removed, using a correlation coefficient threshold of 0.95. The radiomics features were then used to develop the model with logistic regression with least absolute shrinkage and selection operator (LASSO) regularization, penalizing model complexity and selecting the most informative features. After that, a tenfold cross-validation tuning procedure with the area under the receiver operating characteristic curve (AUC) as the optimization metric was used to determine the optimal regularization parameter lambda [23]. The selected features were then used to build a multivariable logistic regression analysis for both radiomics and radiological features to build three different models: radiological features-only, radiomics-only, and combined models. Statistical significance was considered with a *p* < 0.05. Statistical analysis was performed using MedCalc Statistical Software version 22.023 (MedCalc Software, Ostend, Belgium) and MATLAB, version 9.14 (R2024a), The MathWorks, Inc., Natick, Massachusetts, 2024 (https://www.mathworks.com).

### External validation cohort

To evaluate the generalizability of our model, an independent external testing cohort was retrospectively selected. A total of 16 patients who underwent pulmonary resection at Sant’Andrea University Hospital, Rome, Italy were identified using the same inclusion and exclusion criteria as the primary cohort. These patients were acquired between January 2024 and January 2025 at the same hospital using a Philips Brilliance iCT 256-slice scanner with identical acquisition parameters and contrast medium administration protocols. The same contrast agent (Iomeprol 400, Bracco, Milan, Italy) was used, with the dose calculated based on lean body weight, and image acquisition was performed following bolus tracking at the same predefined threshold.

The external cohort was analyzed following the same radiological assessment, segmentation, and feature extraction as the primary cohort.

## Results

### Patients characteristics

From an initial population of 330 patients (215 with adenocarcinoma and 115 with squamous cell carcinoma), 30 (16%) patients were excluded: 20 (66%) patients for severe motion artifacts, and 10 patients (33%) showed at CT nodules too small to be segmented and/or analyzed. Thus, the final population consisted of 300 patients, 116 (39%) females and 184 (61%) males, with a mean age of 68 ± 10 years. Two hundred (66%) patients were affected by adenocarcinoma, and 100 (33%) patients by squamous cell carcinoma. Full patient data are reported in Table 1.

**Table 1:** Study sample characteristics, including histological subtype and patient data

Parameter	Total study sample (n = 300)
*Histological subtype*	
ADC	200
SCC	100
Mean age	68(± 10)
*Sex*	
Females	116
Males	184

Data in parentheses are standard deviation

### Radiological assessment

Of all patients analyzed, 99 (33%) lesions were localized in the left lung (72 adenocarcinoma and 27 squamous cell carcinoma), out of which 41 (41%) lesions were localized in the lower left lobe, 47 (47%) were located centrally (28 adenocarcinoma and 19 squamous cell carcinoma) and 52 (53%) were peripherical (44 adenocarcinoma and 8 squamous cell carcinoma). A total of 167 (56%) patients were found positive for the presence of emphysema (95 adenocarcinoma and 72 squamous cell carcinoma). Full patient data and CT findings are reported in Table 2.

**Table 2:** Radiological features prevalence

	ADC + SCC	ADC	SCC
Number	300	200	100
Location			
Left lower lobe	41 (41%)	30 (73%)	11 (27%)
Left upper lobe	58 (59%)	42 (72%)	16 (28%)
Right upper lobe	133 (66%)	94 (71%)	39 (29%)
Middle lobe	9 (4%)	7 (78%)	2 (22%)
Right lower lobe	59 (29%)	27 (46%)	32 (54%)
Central	145 (48%)	74 (37%)	71 (71%)
Peripheral	155 (52%)	126 (63%)	29 (29%)
Emphysema	167 (56%)	95 (48%)	72 (72%)

Among all the radiological features, two of them showed significant results in the discrimination between ADC and SSC. In particular, the central/peripheral location of the lesion and the presence of emphysema showed an AUC of 0.67 (95% CI 0.61–0.72) and 0.62 (95%CI 0.57–0.68), respectively, with a *p* < 0.0001.

### Radiomics features selection

**Fig. 4:**
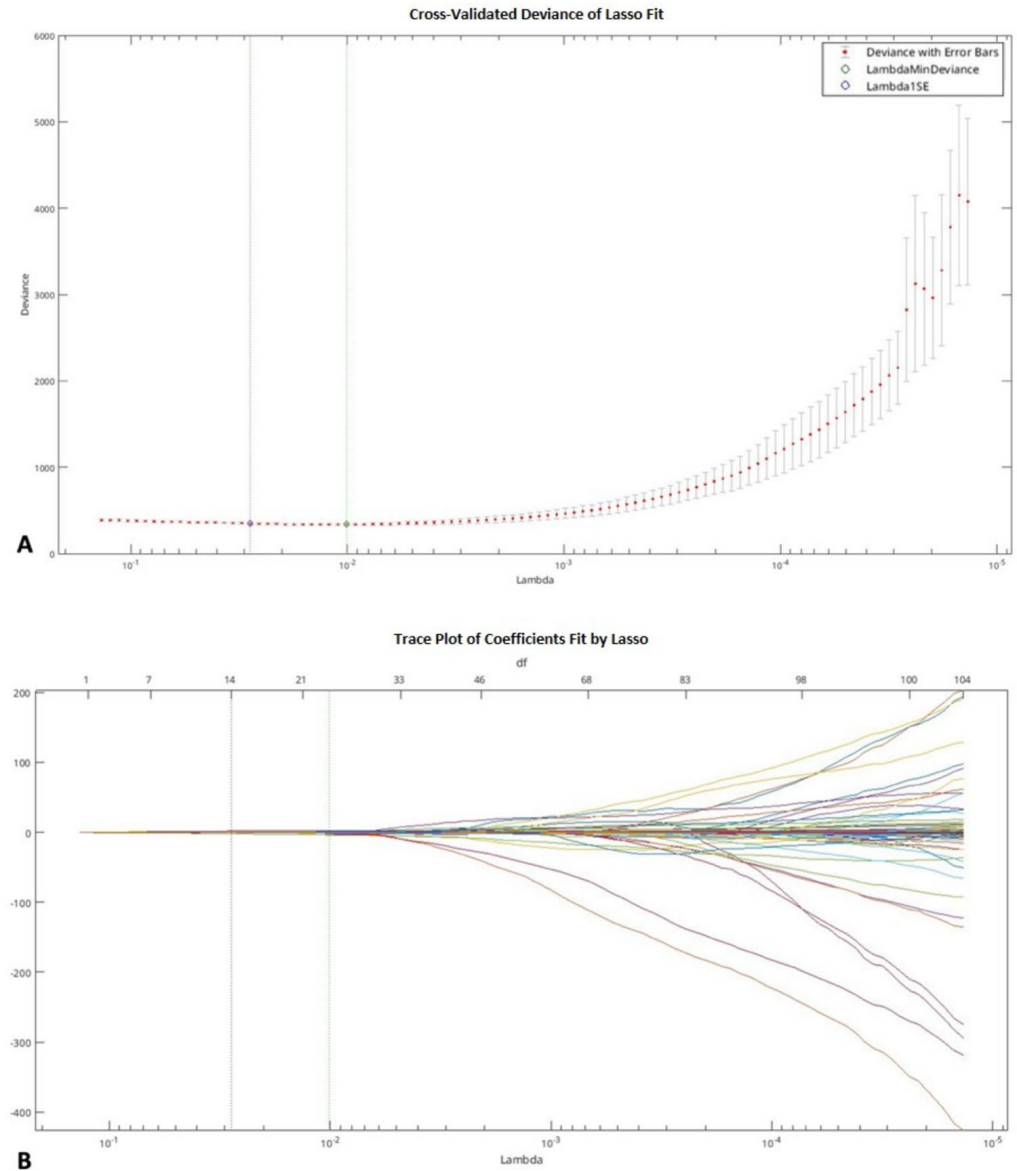
Radiomics feature selection using the least absolute shrinkage and selection operator (Lasso) logistic regression model. (**A**) Turning penalization parameter lambda (λ) using tenfold cross-validation and minimum criterion in Lasso model. The AUC curve was plotted versus log (λ). Log (λ) =  − 4.267, with λ = 0.014 was chosen; (**B**) Lasso coefficient profiles of the radiomics features. The vertical gray line was drawn at the value selected using tenfold cross-validation in (**A**), where the optimal λ yield 20 features with nonzero coefficients

One hundred and seven radiomics features were extracted on chest CT scans from the volumetric segmentation of the lung lesions. The stable features were used for the LASSO regularization and the tenfold cross-validation that selected 20 features (Flatness, MinorAxisLength, Sphericity, Kurtosis, Mean, RootMeanSquared, TotalEnergy, Contrast, Correlation, DifferenceAverage, DependenceNonUniformityNormalized, LargeDependenceHighGrayLevelEmphasis, GrayLevelNonUniformity, GrayLevelVariance, LowGrayLevelZoneEmphasis, SizeZoneNonUniformityNormalized, SmallAreaEmphasis, SmallAreaHighGrayLevelEmphasis, ZoneVariance, Busyness) as reported in Fig. 4.

### Radiological model, radiomics model, and combined model

Multivariable analysis was performed to build the three models: the radiological model, the radiomics model, and the combined model.

The radiological model showed significant results for both central/peripheral location and presence of emphysema with a *p* < 0.001, with OR ranging from 4.83 to 3.34, respectively. The radiological model showed good performance, with an AUC of 0.73 (95% CI 0.68–0.78; *p* < 0.001), with the percentage of correctly classified cases of 72.3%.

**Fig. 5:**
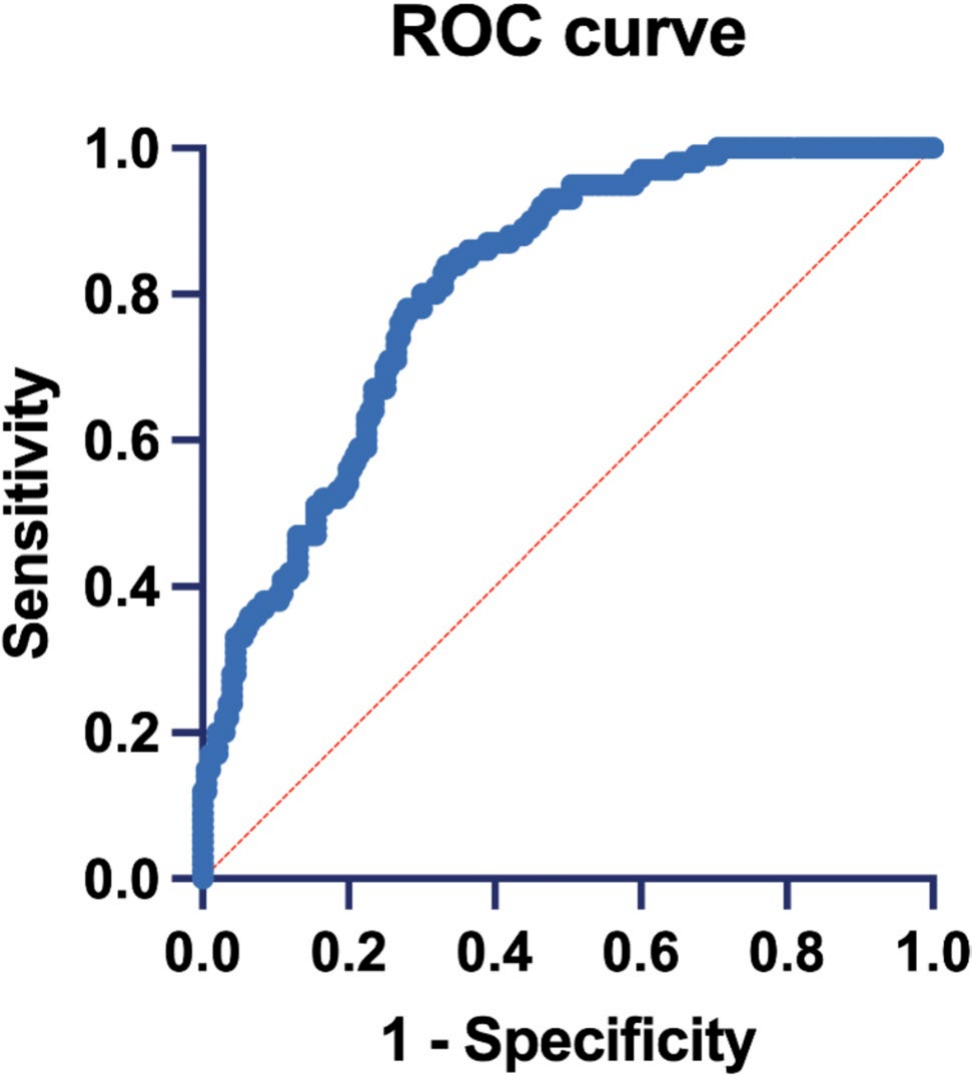
ROC of the radiomic-only predictive model with an AUC of 0.8 and 75.6% of cases correctly classified

For the Radiomics model, among the 20 selected features used for the multivariable analysis, two of them including one Shape (Sphericity), and one GLSZM (SmallAreaHighGrayLevelEmphasis) showed significant results, with P values ranging from 0.03 to 0.001 and OR between 0.013 and 1.12. Multivariable analysis of the radiomics model by including the radiomic features keen to discriminate between ADC and SSC. The radiomic model showed good performance, with an AUC of 0.80 (95% CI 0.75–0.85; *p* < 0.001), with a percentage of correctly classified cases of 75.6% (Fig. 5).

**Fig. 6:**
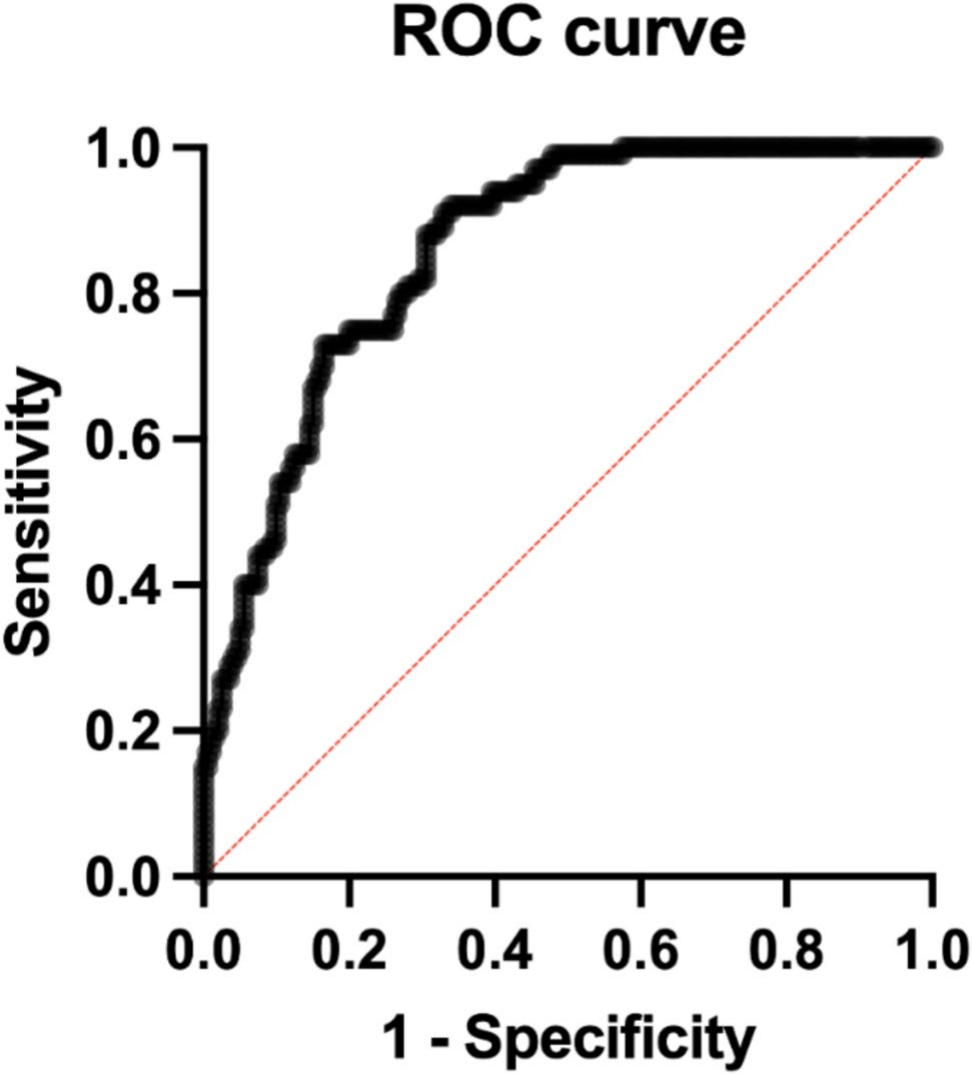
ROC of the combined radiological and radiomics predictive model which achieved the highest performance with an AUC of 0.84 and a percentage of cases correctly classified of 75.3%

Finally, the combined model (radiological features and radiomics features) showed an AUC of 0.84 (95% CI 0.80–0.88; *p* < 0.001), with the percentage of correctly classified cases of 75.3% (Fig. 6).

### External validation

**Fig. 7:**
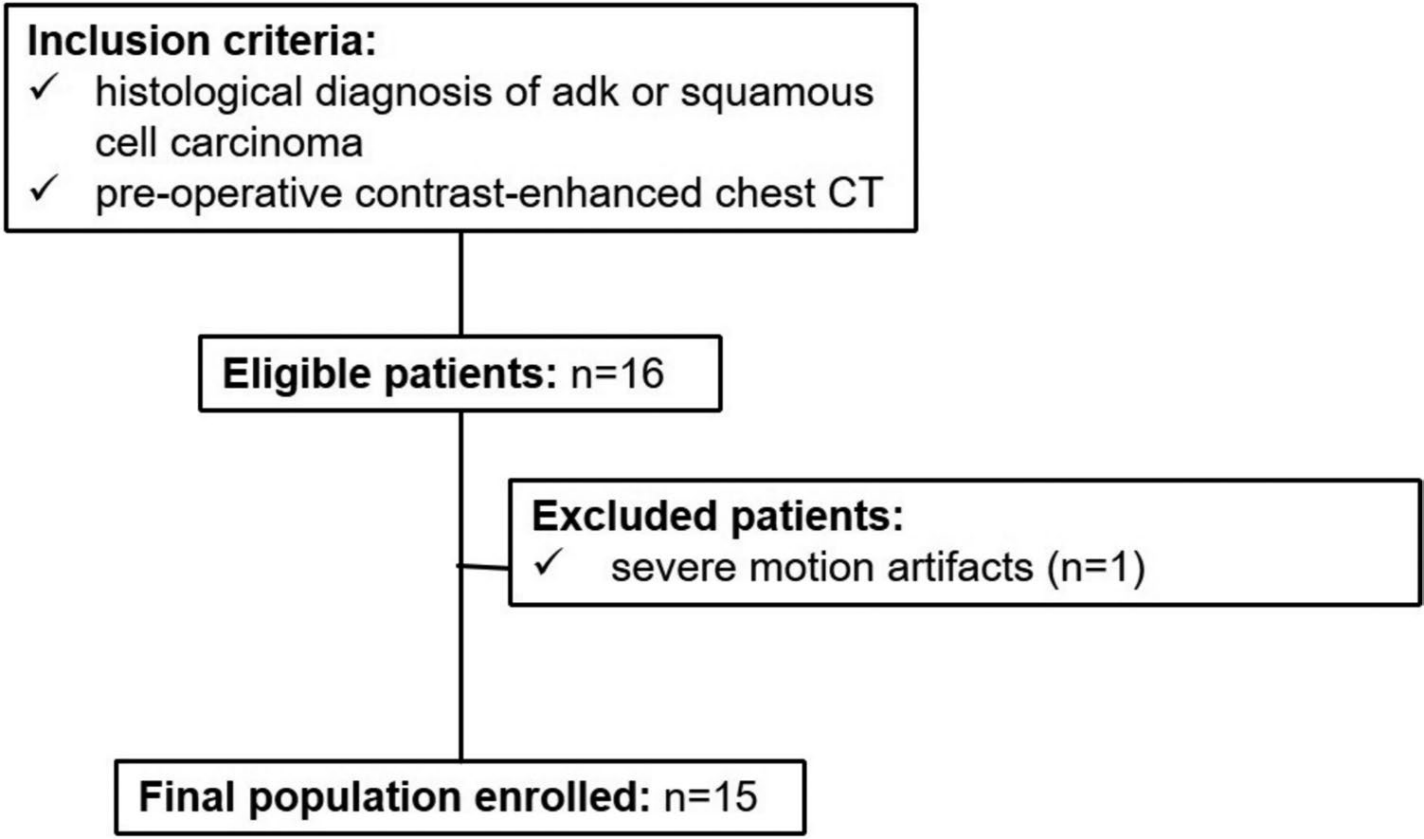
Flowchart shows participant enrollment for external testing

The final population for external validation consisted of 15 patients, of whom 10 (67%) were diagnosed with adenocarcinoma and 5 (33%) had squamous cell carcinoma. One additional patient was initially selected but was excluded due to excessive motion artifacts on CT **(**Fig. 7**)**. This distribution maintained the same disease prevalence rates as observed in the primary study population, ensuring consistency in the validation process.

The previously developed combined model, integrating both radiological and radiomic features, was applied to this external validation cohort to assess its robustness and reproducibility. The model achieved an AUC of 0.78, with a p-value of 0.05, demonstrating its ability to maintain diagnostic performance when tested on an independent dataset.

## Discussion

In this retrospective study, we proposed a predictive model to distinguish lung adenocarcinoma (ADC) from squamous cell carcinoma (SCC) by analyzing the baseline CT scans, including radiological and radiomics features. We initially aimed to integrate radiological and radiomic evaluations to address the inherent limitations of lung biopsy, which is an invasive procedure that can be associated with potential complications, such as infections or pneumothorax, as well as the risk of false-negative results. Furthermore, lung biopsy samples may not fully represent the tumor’s heterogeneity, as they typically capture only a small portion of the tumor [24]. While lung biopsy remains the gold standard in tumor histotype determination [25], our approach sought to complement it by providing a more comprehensive, noninvasive method for tumor evaluation.

Concerning the radiological parameters, the clinical T and N staging, the central or peripheral cancer localization, the lung lobe affected, and the presence of lung emphysema were assessed. Radiomics analysis was performed by extracting quantitative features from the volumetric segmentations, which were performed manually on the primary lung cancers. Then, three different models were developed, the radiological model, the radiomics model, and the combined model. Crucially, the validity of these models was tested on the external validation cohort that achieved a *p*-value of 0.05, with a trend close to the significance with an AUC that confirm the robustness and generalizability beyond the initial dataset.

Our radiological findings suggested that most SCCs were associated with emphysema and were peripheral, just as demonstrated in a previous descriptive study proposed by the group of Kunihiro Y. [19]. Looking at the radiomic analysis of lung lesions, several quantitative features resulted in significant differences between the two groups. These differences led us to build a predictive model to identify the histology from baseline CT scans, yielding a good performance, in line with some previous similar studies that investigated the role of radiomics in classifying lung cancer based on CT, MRI, or PET/CT scans [26–30]. Among these studies, the most relevant results in differentiating ADC and SCC were achieved by the groups of Jing L. [26] and Zhiyong C. [28]. The first group [26] proposed to build a classifying radiomic model both to differentiate ADC, SCC, and large cell carcinoma and to assess clinical staging by analyzing the preoperative CT scan. The best results were reached for the prediction of clinical staging, with an AUC of around 0.9, while only moderate results were demonstrated for histological classification, with an AUC of 0.7. In a separate study, the group of Zhiyong C. [28] tested the predictive role of radiomics in distinguishing ADC from SCC by using the segmentation of pre-therapy dual-energy CT scans and built three different predictive models: clinical-only, radiomics-only, and combined models. The combined model outperformed the radiomics- and clinical-only models, achieving the best AUC of around 0.85. The combined model outperformed both the radiomics-only and clinical-only models, achieving the highest AUC of around 0.85. A distinguishing feature of this study was the selection of clinical data, which included epidemiological information, sex, smoking status, metastatic disease, and carcinoembryonic antigen levels while excluding conventional medical image evaluations. A different approach to identify ADC from SCC was proposed by Zhang J. et al. [30]; they investigated the role of radiomics by extracting quantitative features from the CT segmentations of brain metastases. The results were promising in terms of AUC, supporting the concept that quantitative imaging could serve as a valuable predictive tool, helping physicians to better understand cancer phenotypes and offering an alternative to biopsy, particularly in challenging cases or with fragile patients.

All the aforementioned studies enhanced the role of quantitative imaging in providing a correct overview of lung histology in the pre-therapy setting with promising and brilliant data. Nevertheless, analyzing these studies, the major limitations are the limited number of patients and unbalanced populations, which could lead to relevant limitations in almost all studies and even amplified in all radiomic models. In general, the best results in terms of performance were achieved by integrating radiomics with clinical data, outperforming radiomics or clinical models, but there still needed to be a concordance in the selection of clinical data. The “clinical” term included a very large number of different parameters, starting from epidemiological to clinical staging. This is a crucial aspect that highlights the need for large multicentric and prospective studies, which could standardize the methodology to make the quantitative approach feasible and reproducible in clinical practice.

In the new era of personalized medicine and target therapy, in which understanding all the facets of neoplasms is essential to outline the therapeutic strategy tailored per patient, our study could have a relevant impact. Historically, the SCC has a worse prognosis in terms of survival rate and clinical staging at the diagnosis [31]. It recently emerged that the neoadjuvant approach in SCC with chemotherapy in association with immunotherapy could change the natural history of SCC in terms of pathological complete response, progression-free survival, and overall survival [32, 33]. These data supported the idea that a pre-treatment evaluation of cancer phenotype is essential to provide the patient with the best treatment option, as well as the need to reduce any risks linked to the intrinsic bias of conventional biopsy [34]. In such a scenario, radiomics might have a future role in pre-treatment lung cancer evaluation. Nevertheless, it is not right to think of radiomics as a panacea but only as a supporting tool for clinicians, to have a more accurate overview of tumor microenvironmental and microarchitectural before starting therapy, which should be permanently integrated with the clinical data.

It is important to acknowledge the inherent limitations associated with radiomics analyses. Specifically, the relatively small sample size may have impacted the robustness of the derived features and model performance. Additionally, the retrospective nature of the study introduces potential biases, which should be considered when interpreting the results. Lastly, the intrascanner or interscanner variability should be evaluated as a confounding factor that could alter the final results and the reproducibility of the analysis [35]. Looking at our limitations, we tried to overcome the problem of a small sample size, by enrolling up to 300 patients. Furthermore, our population was quite balanced, including 200 ADC and 100 SCC, maintaining the epidemiological incidence of each histology [1]. Among the limitations, it is also important to recognize the small sample size of the validation cohort, which obtained a nearly significant result that confirms the trend of the test cohort but would have benefited from a larger number of subjects.

A critical future direction involves the establishment of a prospective, multi-center cohort to rigorously validate the combined model. This prospective design will mitigate biases inherent in retrospective analyses and facilitate the assessment of the model’s generalizability across diverse patient demographics and clinical settings, thereby strengthening its clinical utility.

To conclude, in this study we developed a radiological-radiomics model based on baseline CT scans to differentiate between adenocarcinoma (ADC) and squamous cell carcinoma (SCC), outperforming both radiomics- and radiological-only models. This quantitative approach might provide an accurate and noninvasive method to determine lung cancer histotypes at diagnosis, assisting the oncologists in clinical decision-making, and reducing any risk of uncorrected diagnosis due to intrinsic limitations of conventional biopsy.

## Abbreviations

NSCLC: Non-small-cell lung cancer
ADC: Adenocarcinoma
SCC: Squamous cell carcinoma
